# The Effects of Climate Seasonality on Behavior and Sleeping Site Choice in Sahamalaza Sportive Lemurs, *Lepilemur sahamalaza*

**DOI:** 10.1007/s10764-018-0059-1

**Published:** 2018-09-11

**Authors:** Isabella Mandl, Marc Holderied, Christoph Schwitzer

**Affiliations:** 1 School of Biological Sciences, University of Bristol, Bristol, BS8 1TQ UK; 2 Bristol Zoological Society, Bristol Zoo Gardens, Clifton, Bristol, BS8 3HA UK

**Keywords:** Adaptation, Behavior, Nocturnal, Seasonal, Strepsirrhines

## Abstract

Temperature, rainfall, and resource availability may vary greatly within a single year in primate habitats. Many primate species show behavioral and physiological adaptations to this environmental seasonality, including changes to their diets and activity. Sahamalaza sportive lemurs (*Lepilemur sahamalaza*) inhabit the northwest of Madagascar and have been studied only during the dry, colder period of the year. We investigated potential effects of climate seasonality on this species by collecting behavioral data between October 2015 and August 2016, encompassing both the warmer wet and the colder dry seasons. We collected 773.15 hours of behavioral data on 14 individual sportive lemurs to investigate year-round activity budgets, ranging behavior, and sleeping site locations. Additionally we recorded temperature and rainfall data at our study site to describe the environmental conditions during the study period. The study individuals significantly decreased their time spent traveling and increased their time spent resting in the dry season compared to the wet season. Although home range size and path lengths did not differ over the study period, sleeping locations were significantly different between seasons as the lemurs focused on more confined areas in colder periods. Overall, the results indicate that Sahamalaza sportive lemur behavior varies with season, in line with reports for other primates.

**Electronic supplementary material:**

The online version of this article (10.1007/s10764-018-0059-1) contains supplementary material, which is available to authorized users.

## Introduction

Most primate species inhabit tropical and subtropical regions (Myers *et al.* 2000; Wilson 1988). These regions are characterized by high precipitation and, to varying extent, seasonality. Seasonality is commonly defined as “the occurrence of certain obvious biotic and abiotic events, or groups of events, within a definite limited period, or periods, of the astronomic (solar or calendar) year” (Lieth 1974, p. 5). The degree of seasonality depends on latitude, as habitats closer to the equator do not show pronounced fluctuations in abiotic factors, such as rainfall and temperature (Addo-Bediako *et al.* 2000; Stevens 1989; van Schaik and Pfannes 2005). In these regions, rainfall occurs year-round or in multiple shorter rainy seasons. These short periods merge into one longer rainy season as one moves away from the equator (van Schaik and Pfannes 2005). Dry seasons, which are usually defined as the number of subsequent months in which rainfall is <100 mm/mo (Hemingway and Bynum 2005; van Schaik and Pfannes 2005), therefore increase in length with increasing latitude, resulting in more pronounced seasonal differences in climate (van Schaik and Pfannes 2005). Similarly, year-round fluctuations in photoperiod increase with increasing latitude (Hill 2005).

Recurring oscillations in rainfall, temperature, and photoperiod may affect biotic factors such as plant phenology (Lieth 1974). The resulting variation in food and water availability may influence and shape aspects of primate ecology and behavior by affecting year-round activity budgets, diet composition, and habitat use (Brockman and van Schaik 2005). Seasonality can further influence the timing of physiological processes such as reproduction (Brockman and van Schaik 2005; Rasmussen 1985) and can affect primates indirectly via varying levels of predation pressure throughout the year (Gursky and Nekaris 2007; Irwin *et al.* 2009; Karpanty and Wright 2007; Mitani and Watts 2005; Rasmussen 2005).

Correlations among temperature fluctuations, changes in day length, and food-rich (or scarce) periods make it difficult to identify a single underlying mechanism driving primate behavioral adaptations (Brockman and van Schaik 2005) and limit our ability to predict the effects of climate change on primates. Generally, in times of low resource abundance, primates switch diets, increase their ranging, or attempt to save energy by reducing their overall activity (Ganzhorn *et al.* 2003). However, resource abundance can be coupled with abiotic variables such as temperature and rainfall (Tutin and Fernandez 1993; van Schaik *et al.* 1993), which themselves can affect primate behavior (Brockman and van Schaik 2005). Therefore, it is likely that primate ecology and behavior is shaped by an interplay of all these factors, as suggested for lemurs (Wright 1999). Madagascar lies in the most southern tropical latitudes and is characterized by varying degrees of seasonality (Richard *et al.* 2002; Wright 1999; van Schaik and Pfannes 2005): the central highlands as well as the western and northern regions are marked by long dry seasons, while the southern region is characterized by very little overall rainfall; high precipitation is common throughout the year in the eastern region of the island (Ganzhorn *et al.* 2001; Tattersall and Sussman 1975).

Across Madagascar, lemurs have evolved multiple adaptations to conserve energy in environmentally stressful times (such as cold, dry seasons): most species show low basal metabolic rates and highly seasonal reproduction while torpor and hibernation are common among the smaller lemuroids (Kappeler and Ganzhorn 1993; Wright 1999). Thermoregulatory and energy-conserving behavior often occurs in areas with a prolonged dry season—a period in Madagascar marked by lower temperatures, little precipitation, and food and water scarcity (Sato *et al.* 2014; Wright 1999). Diurnal and cathemeral lemur species rest more, travel less, and increase sunbathing behavior during the dry season, particularly when ambient temperatures are low, e.g., in collared brown lemurs (*Eulemur collaris*: Campera *et al.* 2014; Donati *et al.* 2010), diademed sifakas (*Propithecus diadema*: Irwin 2014), brown lemurs (*E. fulvus rufus*: Sato 2012), ring-tailed lemurs (*Lemur catta*: Simmen *et al.* 2010), and ruffed lemurs (*Varecia rubra*: Vasey 2005; *V. variegata variegata:* Morland 1993). Decreased activity levels are not the only strategy lemurs use: brown lemurs (*E. fulvus rufus*) inhabiting the highly seasonal northwest of Madagascar show a combination of dietary and habitat changes in response to fruit scarcity during the dry season (Sato 2013; Sato *et al.* 2014).

For small-bodied, nocturnal lemurs, seasonality in climate may impose even greater constraints as temperatures during the dry season drop significantly at night (Aujard *et al.* 1998). To cope with this environment, members of the family Cheirogaleidae often hibernate during cooler periods of the year (Blanco and Rahalinarivo 2010; Dausmann *et al.* 2004; Fietz and Dausmann 2006; Kobbe *et al.* 2014; Ortmann *et al.* 1997; Schmid 2000; Schülke and Ostner 2007). Nocturnal species that do not hibernate or show seasonal torpor, however, exhibit similar behavioral adaptations to diurnal lemurs: sportive lemurs of the family Lepilemuridae rest more and travel less during the dry, colder season (Dröscher and Kappeler 2014; Dröscher *et al.* 2016; Ganzhorn 1993; Hladik and Charles-Dominique 1974; Nash 1998). Their reproduction is highly seasonal, and births are timed around the end of the dry season (Hilgartner 2006). These small (600–1200 g; Mittermeier *et al.* 2010), arboreal primates are highly folivorous (Dröscher and Kappeler 2014; Hladik and Charles-Dominique 1974; Hladik *et al.* 1980; Seiler *et al.* 2014). Recent studies show that, although many sportive lemur species occur in deciduous forests where leaf availability fluctuates, dry seasons may not necessarily pose energy constraints in terms of food scarcity, but rather induce cold stress (Dröscher *et al.* 2016; Dröscher and Kappeler 2014; Ganzhorn 2002). As a behavioral response, sportive lemurs decrease their activity levels and ranging distance and increase resting times during the colder period (Ganzhorn *et al.* 2004; Nash 1998). White-footed sportive lemurs (*Lepilemur leucopus*) even increase food intake and time spent feeding during the colder period, possibly to compensate for higher energetic demands due to colder temperatures (Dröscher and Kappeler 2014; Dröscher *et al.* 2016).

As most sportive lemur species were described only in the past decade (Andriaholinirina *et al.* 2006; Craul *et al.* 2007), we have limited knowledge of their behavior and ecology in comparison to what is available for other primate taxa. This includes the Critically Endangered Sahamalaza sportive lemur, *Lepilemur sahamalaza* (the name was changed recently from *L. sahamalazensis*: Andriaholinirina *et al.* 2017), inhabiting the last remaining forests of the Sahamalaza Peninsula in northwest Madagascar. Previous studies that collected data exclusively during the colder, dry season indicate that Sahamalaza sportive lemurs are overall very inactive, with long resting bouts between short bursts of activity (Seiler *et al.* 2015) and that they adjust their behavior to rainfall, e.g., by switching between sleeping sites (Seiler *et al.* 2013). However, owing to the high seasonality in this region, with dry seasons lasting up to 6 mo each year (Volampeno *et al.* 2011), the results may not provide a complete understanding of Sahamalaza sportive lemur behavior.

We conducted year-round nocturnal behavioral observations of 14 Sahamalaza sportive lemurs to record activity budgets. We hypothesized that the species’ activity budget and sleeping site locations differ between the wet and dry season. If the seasonality of the habitat, measured as temperature and rainfall fluctuations throughout the year, is an environmental stressor, then we predicted that Sahamalaza sportive lemurs would show behavioral adaptations to this variable environment by adjusting activity budgets, ranging behavior, and sleeping site choice. Specifically, we predicted that Sahamalaza sportive lemurs would rest more and travel less in colder periods. We further predicted that home range size would decrease as a reflection of a decrease in general activity but did not predict that the home range location would move between the wet and the dry season. Finally, we investigated the effects of seasonality on sleeping site location, predicting a shift between months with rainfall and those without.

## Methods

### Study Site

**Fig. 1:**
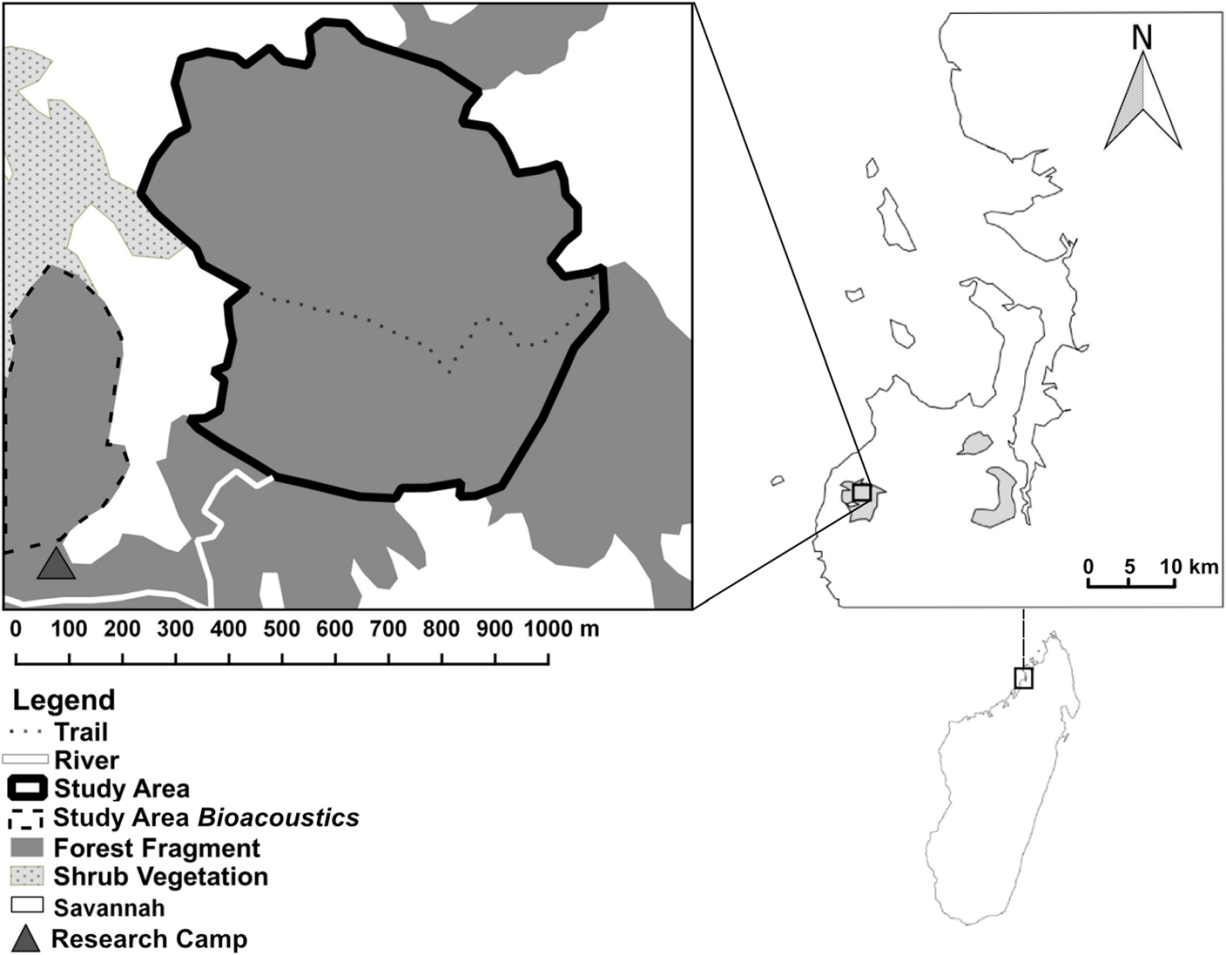
Location of the study site in Ankarafa Forest, Sahamalaza National Park (gray shaded area, first insert), Sahamalaza Peninsula, northwest Madagascar. The study area (black outline, second insert) is ca. 50 ha in size. Data were collected between October 2015 and August 2016.

We conducted the study in Ankarafa Forest in Sahamalaza–Iles Radama National Park, in northwestern Madagascar (Fig. 1). Ankarafa Forest is the most western forest patch in the protected area located between 13°52'S and 14°27'S as well as 45°38E and 14°46'E and is characterized by a mix of dry deciduous and Sambirano rainforest vegetation structures with a canopy up to 25 m in height, as is typical for Malagasy lowland forests (de Gouvenain and Silander 2003; Dumetz 1999; Grubb 2003; Volampeno *et al.* 2013). The forest consists mainly of regenerated forest with some old growth vegetation remaining (Seiler *et al.* 2013), and human activities and anthropogenically caused fires have influenced the vegetation structure: differently degraded patches occur throughout the forest. In addition, nearly a quarter of the forest is composed of exotic and invasive species, such as mango trees, *Mangifera indica*, and nonnative bamboo, *Bambusa* sp. (Schwitzer *et al.* 2007; Volampeno *et al.* 2013).

We collected data for 10 mo between October 2015 and August 2016 covering the wet season (October–March) and the dry season (April–September). We further divided the seasons into early and late subseasons to improve the resolution of the data (Table I). We based this division on preliminary weather data (early subseasons incorporate the transition periods) and on Sahamalaza sportive lemur reproductive patterns (birth, premating, mating, and postmating) based on previous observations and preliminary data (Ruperti 2007; Seiler *et al.* 2015). Infants were born between late September and early November, while F. Ruperti observed mating in May and early June. During the study period we recorded daily minimum and maximum temperatures, measured with digital thermometers (TFA Dostmann, Wertheim, Germany), and measured daily rainfall using a simple rain gauge.

**Table I:** Definitions of seasons and subseasons used in a study of Sahamalaza sportive lemurs in Ankarafa Forest, northwest Madagascar, between September 2015 and August 2016

	Season			
	Wet		Dry	
Subseason	Early wet	Late wet	Early dry	Late dry
months:	October/November/December	January/February/March	April/May/June	July/August/September
Reproductive period	Birth	Premating (lactation)	Mating	Postmating (gestation)
months:	September–November	November–March	March–June	July–September

We did not collect data in September (late dry) and March (late wet)

### Study Subjects

We captured 14 Sahamalaza sportive lemurs between September and October 2015 in the study forest and fitted them with radio-collars (3.5 g, Biotrack, Wareham, UK). Four individuals vanished during the data collection period: we assumed three deaths were due to predation as we found carcasses or remains; we could not account for one disappearance. Three individuals vanished at the beginning of the early dry season and the fourth at the end of the study. Unless stated otherwise, we included data for all available individuals in the analyses.

### Annual Activity Budget and Home Ranges

We conducted behavioral observations on the radio-collared individuals throughout the study. Two teams, consisting each of three observers, followed two individuals simultaneously 18:00 h–24:00 h; each team used a SIKA radio-tracking receiver (Biotrack, Wareham, UK) and 3-element Yagi antenna (Biotrack Wareham, UK). In a pilot study, I. Mandl calculated activity budgets from 327.7 h of continuous behavioral observation on six individual Sahamalaza sportive lemurs between July and October 2013. Comparing the percentage of time spent on each individual behavior (see Table II) with paired *t*-tests for the first (18:00 h–24:00 h) and the second half of the night (0:00 h–06:00 h) revealed no statistically significant differences (resting: *t*_(5)_ = 0.97, *p* = 0.39; feeding: *t*_(5)_ = 0.9, *P* = 0.40; grooming: *t*_(5)_ = −0.7, *P* = 0.52; locomotion: *t*_(5)_ = 1.2, *P* = 0.21, and not visible: *t*_(5)_ = 1.5, *P* = 0.19). We therefore collected behavioral data only during the first half of the night. The observers followed the focal individuals using headlamps and torches and dimmed these lights if the animals were close. We recorded behaviors continuously, as described in the ethogram (Table II), giving a detailed activity budget for each individual. We recorded GPS points with a handheld GPS (GPSMAP 60CSx, Garmin Ltd., Schaffhausen, Switzerland) at each tree the focal individual visited during observation. Although observations of behaviors were often difficult because of the dense canopy, the sportive lemurs did not show flight behaviors typical of other primates and occasionally approached the observers as close as 1 m to settle and feed. The observers aimed to avoid disturbance to natural behaviors where possible.

**Table II:** Ethogram of recorded behaviors during nocturnal observations of Sahamalaza sportive lemurs in Ankarafa Forest, northwest Madagascar, between October 2015 and August 2016

Behavior	Description
Resting Vigilant	The lemur is stationary but alert, looking around and directing its gaze in various directions.
Resting	The lemur sits on a support, is not alert, and directs its gaze in one direction, eyes half-closed or closed.
Feeding	The lemur is handling food and eats, chewing visibly or audibly. If the lemur was partially or wholly out of view, we ascertained feeding behavior by the characteristic rustling and dropping of half-eaten food items, such as leaves.
Grooming	The lemur is licking or scratching its fur.
Locomotion	The lemur moved, by walking, climbing, or jumping, over a distance of >50 cm. If the lemur was partially or wholly out of view, we ascertained locomotion by movement of branches and leaves at the animals’ location.
Other	Behaviors not described above, including social interactions, vocalizing, and infant care.
Not visible	The lemur is not clearly visible and we cannot observe its behavior.

### Sleeping Sites

**Fig. 2:**
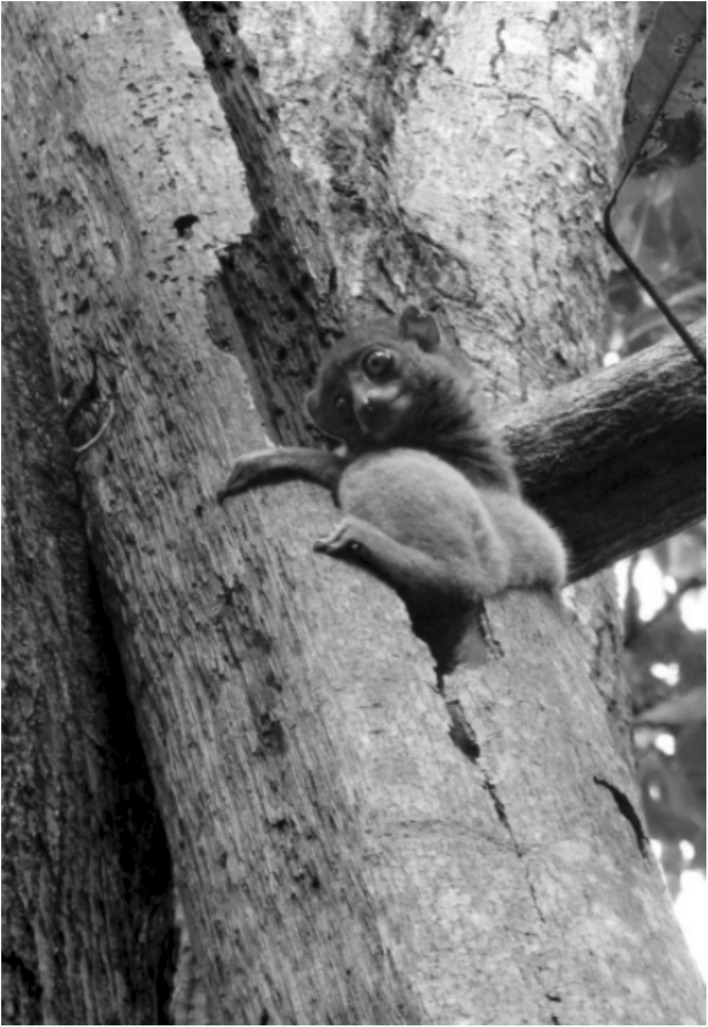
A Sahamalaza sportive lemur (F3) in Ankarafa Forest, Sahamalaza National Park, northwest Madagascar resting at a sleeping site during the day in July 2016.

We visited the study individuals at their sleeping sites three times a week during the study (Fig. 2). We located individuals at their sleeping sites and identified them with the help of their radio-collars. We recorded a GPS point for the sleeping site with a handheld GPS if we could locate it clearly. If animals were not visible, we ascertained their location using the signals of the radio-collars and triangulation. We recorded the visibility of the individual (visible/not visible) at each site. The time of day we recorded sleeping site locations varied throughout the study period, but we assume that this did not influence our data, as Sahamalaza sportive lemurs do not usually move or change sleeping sites during the daytime (Ruperti 2007; Seiler *et al.* 2013). If we could not identify the sleeping tree clearly, we did not take a GPS point.

### Analysis

#### Temperature and Rainfall

We calculated the mean minimum and maximum temperatures for each month and each subseason and compared them between subseasons using a Kruskal–Wallis analysis of variance. We calculated total rainfall for each month. Rainfall occurred mainly during the daytime but we terminated five nightly observations in February early because of excessive rainfall. We excluded these from our analysis. We, therefore, did not examine the effects of rainfall on behavior. We considered rainfall only in analysis of path lengths for nights where rainfall was light enough to allow for full behavioral observations.

#### Activity Budget, Home Range Size, and Path Length

We collected 773.15 h of behavioral data between October 2015 and August 2016. We determined activity budgets using only the time the animals were clearly visible (Table III). We calculated the percentage of time an individual spent on each behavior for each subseason. To test whether season affected activity budgets we computed linear mixed models (LMMs) for each behavior, the response variables being the percentages of time engaged in the behavior, the fixed effects being subseason (early wet, late wet, early dry, late dry) and sex (male/female), and an interaction between the two. We set individual ID as a random effect to account for interindividual differences. We also controlled for differences in behavior due to similarities in habitat structure by including a random effect of forest area. We classified individuals whose home ranges overlapped to any degree as living in the same area, resulting in five forest areas). We added 0.5 to all values to eliminate analysis problems arising from 0 values and log-transformed the response variables to achieve normality.

**Table III:** GPS points and total observation time for 14 radio-collared Sahamalaza sportive lemurs observed in Ankarafa Forest, northwest Madagascar between October 2015 and August 2016

Individual	Total observation time (h) including time not visible	Observation time when visible (h)	Number of GPS points
F1	62.75	23.34	398
F2†	38.75	17.78	287
F3†	51.75	31.92	459
M4†	22.00	12.03	301
F5	64.75	33.47	215
F6	59.45	34.42	126
F7	65.00	35.68	349
M8	64.25	27.49	319
F9	78.15	50.40	493
M10†	24.50	13.18	106
M11	63.75	32.89	428
M12	61.75	37.60	587
F13	60.15	26.40	407
M14	56.15	24.76	200
Total	773.15	401.47	4675

†Individuals that died or vanished during the study

To compute home range sizes, we visualized all GPS points collected during behavioral observations using Quantum GIS (ver. 2.14.0, QGIS Development Team). We excluded eight points, as they were clearly not within the range of the respective individuals (e.g., lying up to 1 km outside the forest border), indicating measurement errors. We used the remaining GPS points to compute kernel density estimation (KDE) distributions: home ranges that illustrate a percentage likelihood of an animal residing in a given area based on the GPS relocations (Worton 1989). We calculated 50 and 99% KDEs for each individual using a least-squares cross validation (LSCV) bandwidth selector. We chose 99% KDEs rather than the commonly used 95% density estimation because LSCV may undersmooth estimations of small home ranges of <1 ha, giving very conservative results (Blundell *et al.* 2001; Seaman and Powell 1996; Steury *et al.* 2010). We did not follow a fixed time schedule but collected GPS points for every tree we saw the lemurs visit, resulting in varied temporal autocorrelation and different sample sizes. As the study individuals often rested in the same tree for multiple hours, a fixed sampling regime would have resulted in datasets consisting in large part of duplicates, introducing biases in utility distributions (Katajisto and Moilanen 2006). However, while introducing a fixed sampling regime to decrease biases in how home ranges are used is recommended (de Solla *et al.* 1999), calculating home range size via KDE does not necessarily require independent data points (Blundell *et al.* 2001; de Solla *et al.* 1999; Swihart and Slade 1997). We therefore focused on calculating home range size from the available data, rather than interpreting home range use, but acknowledge that the limited sample sizes may have decreased home range size estimates (see Electronic Supplementary Material [ESM] Fig. S1 for plots of cumulative home range size that illustrate a steep increase in size estimate with fewer nights of data collection).

We compared mean home range size between the wet and the dry season using paired Students *t*-tests (de Winter 2013). We also calculated the percentage of overlap of the 50% core KDEs (the area an animal is most likely to be found in 50% of the time) in the wet and dry seasons to determine if the study individuals changed their centers of activity over time. We excluded three individuals from this analysis, as they disappeared at the beginning of the dry season and we did not have complete home range data for them.

We could not compare the size of the home ranges for each subseason because of limitations of the datasets. We therefore focused on the daily path length to describe variation in ranging behavior. We calculated daily path length as the distance traveled in meters for each individual for each night of full behavioral observations (18:00 h–24:00 h) using the GPS points collected during those nights with the Points-to-Paths plugin in Quantum GIS (Hiatt 2015). We investigated the effect of temperature and subseason on path length over the year with a LMM, setting minimum temperatures measured on days for which path length data were available, sex of the individual, and subseason as fixed effects. As rainfall affects primate ranging behavior (e.g., Ganas and Robbins 2005), we included rainfall (nights with >5 mm rainfall vs. those with <5 mm rainfall) as a fixed effect. However, this analysis encompasses only nights in which rainfall was light enough to allow full behavioral observations. We also included individual ID and forest area as random effects. We considered only minimum temperatures in the analysis, as these were recorded at night during the active period of the sportive lemurs and may thus have directly influenced activity.

### Sleeping Sites

We calculated the percentage of days each individual was visible at its sleeping sites for each subseason, using the total number of days we recorded the individuals at their sleeping sites. Although we could determine the exact sleeping tree for most days, the study individuals were often not visible, as they often hid in tree holes or foliage, making it impossible to quantify sleeping site types. We plotted the collected GPS points of sleeping sites onto each individual’s home range for each season to compare 1) the location and 2) the spread of sites. We calculated the spread of sleeping sites by determining the distance of each sleeping site to the mean GPS location of sleeping sites, the centroid (= standard distance). The centroid is calculated as the mean latitude/longitude values of sleeping sites for each subseason and individual. Then we investigated the effect of season on the log-transformed standard distances using an LMM, including individual ID and annual home range size as random factors to account for interindividual variation and differences in distance caused by home range size.

We performed all statistical analysis using R (ver. 3.3.1, R Core Team) using the packages MASS (Venables and Ripley 2002), sp. (Pebesma and Bivand 2005), and raster (Hijmans 2016). We computed home range calculations using the package adehabitatHR (Calenge 2006) and produced all LMMs with the package lme4 (Bates *et al.* 2015). For all LMMs, we explored the data to ascertain that they met the assumptions of the models. We also reviewed diagnostic plots of residuals. Where we made multiple comparisons, we adjusted *p*-values using the Holm–Bonferroni method. We set the significance level to *P* = 0.05 and tests were two-tailed unless stated otherwise.

#### Data Availability

The datasets analysed during the current study are available from the corresponding author on reasonable request.

## Ethical Note

A team of trained veterinarians captured the study individuals during the daytime at their sleeping sites using nets and anaesthetized each individual with 0.1 ml of Zoletil 100 by hand or using a Telinject blowpipe. The team weighed the subjects and took standard measurements. They equipped captured individuals with a microchip (8 mm × 1.4 mm ISO FDXB, Micro-ID, West Sussex, UK) subcutaneously for future identification in case of recapture. We fitted all individuals, eight females and six males, with cable-tie VHF radio-collars that did not exceed 0.7% of their body mass (3.5 g, Biotrack, Wareham, UK). We monitored the captured individuals for ≥6 h before releasing them at the capture site at the onset of their normal nocturnal activity period. We also visited all individuals daily and checked for signs of deteriorating health or problems with the collars. At the end of the study period, we recaptured all remaining individuals using the foregoing methods. All subjects had gained mass over the year and all collared females showed signs of pregnancy, indicating that the collars did not prevent mating and reproduction. The veterinarian team removed the remaining collars successfully and released the individuals back into the wild.

We carried out all procedures with ethical approval from the University of Bristol’s Ethical Review Group (project number UB/14/048), under the revised Animals (Scientific Procedures) Act 1986, and an approval from Madagascar’s Ministère de l’Environnement, de l’Ecologie, de la Mer et des Forets (MEEMF) and Madagascar National Parks (MNP) (permit number 37/16/MEEMF/SG/DGF/DAPT/SCBT).

The authors declare that they have no conflict of interest.

## Results

### Temperature and Rainfall

**Fig. 3:**
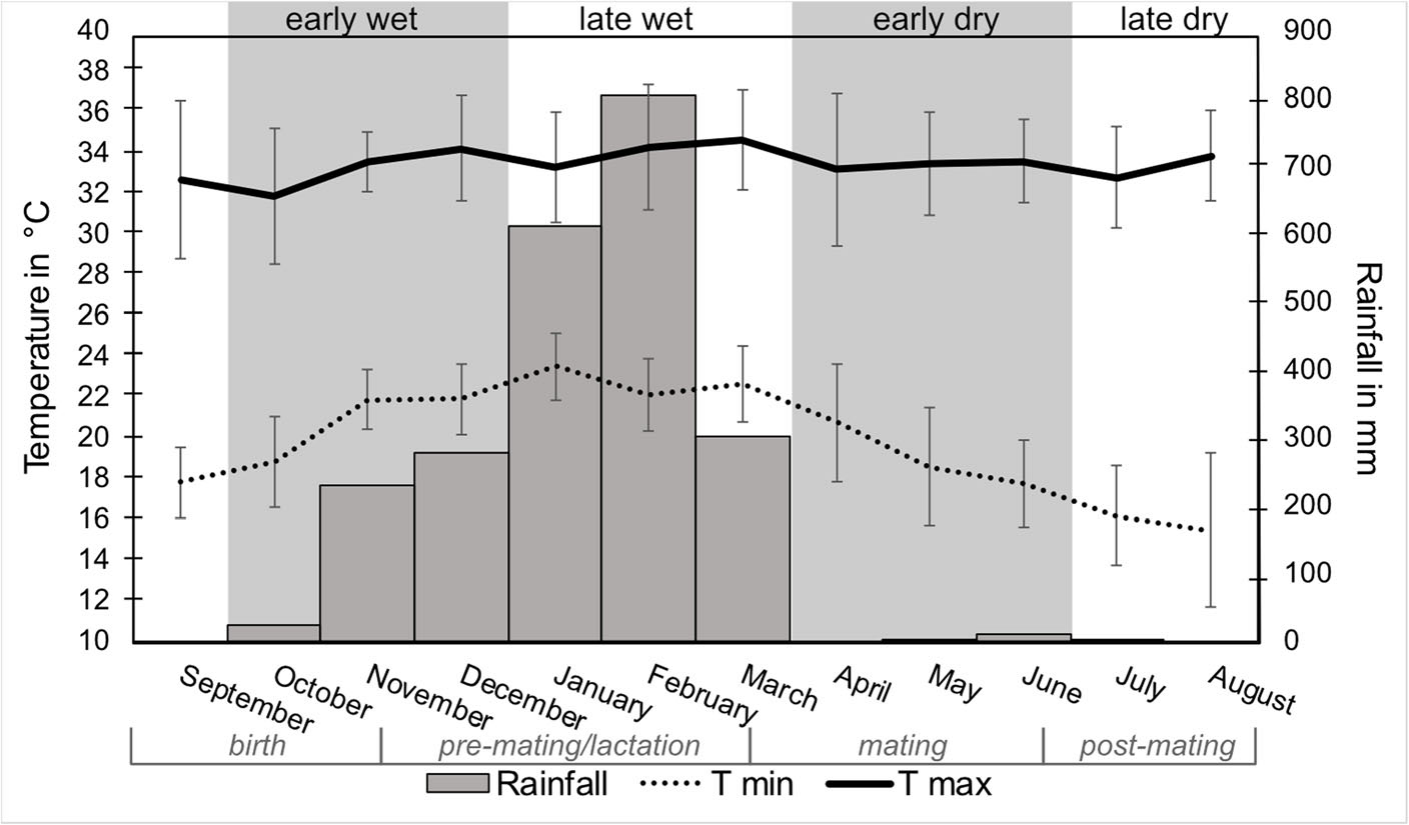
Mean monthly minimum (T min) and maximum temperatures (T max) and total monthly rainfall in Ankarafa Forest, Sahamalaza National Park, northwest Madagascar between September 2015 and August 2016 (*N* = 322 measurements). Error bars represent the standard deviation.

Mean minimum and maximum temperatures and rainfall varied across the study period (Fig. 3). Rainfall was highest in January (612 mm) and February (>800 mm), dropped markedly in March (302 mm), and ceased in April (Fig. 3). Total rainfall over the data collection period was 2252 mm.

**Fig. 4:**
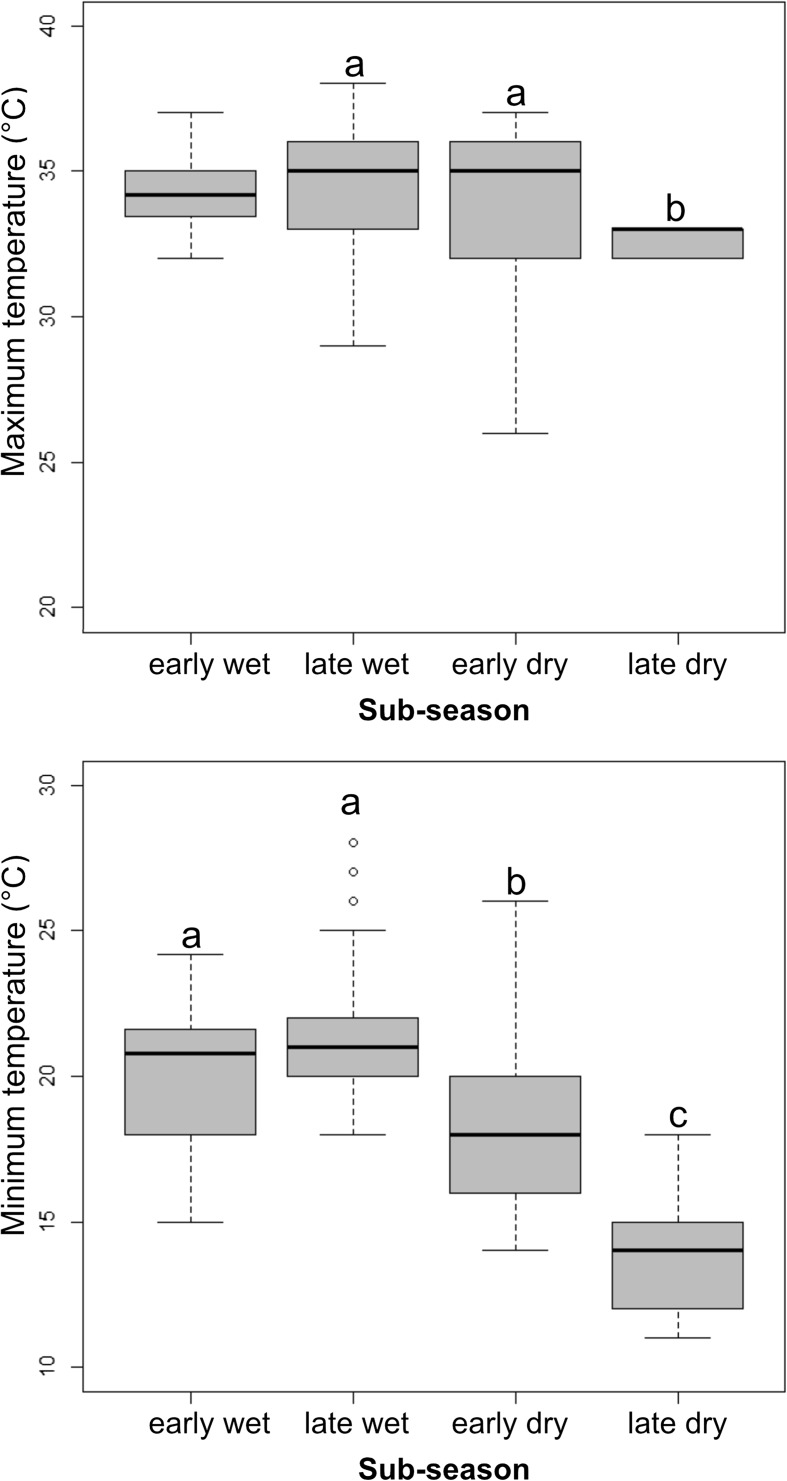
Daily maximum and minimum temperatures (*N* = 322 measurements) in Ankarafa Forest, Sahamalaza National Park, northwest Madagascar, across four subseasons. Letters indicate statistically significant different values at *P* < 0.05, based on a Kruskal–Wallis analysis of variance. NS = nonsignificant. Black bars depict the median, gray boxes the upper and lower quartiles, and whiskers the range.

Both minimum and maximum temperatures showed significant differences between the subseasons (Kruskal–Wallis test: *T*_min_: *H* = 146.21, df = 3, *p* < 0.01; *T*_max_: *H* = 4.753, df = 3, *P* < 0.01). Pairwise Mann–Whitney *U* tests revealed that maximum temperature was significantly lower in the late dry season than in the early dry (*z* = −1.91, *P* = 0.05) and the late wet season (*z* = −3.04, *P* = 0.002), despite a drop in maximum temperature during the wet season. Minimum temperature did not differ significantly between the early wet and the late wet seasons, but all other comparisons showed statistically significant differences (early wet–early dry: z = −4.91, *P* < 0.01; early wet–late dry: *z* = −7.67, *P* < 0.01; early dry–late dry: *z* = −7.01, *P* < 0.01) as the temperature decreased over time (Fig. 4).

### Seasonal Effects on Activity Budget

The study subjects spent a mean of 50% of their active time Resting Vigilant in all subseasons. Time spent on all other behavioral categories varied across the year (Table IV).

**Table IV:** Activity budget (in %) of 14 Sahamalaza sportive lemurs recorded in Ankarafa Forest, northwest Madagascar between October 2015 and August 2016, across all subseasons (*N* of individuals = early wet: 14, late wet: 14, early dry: 12, late dry: 11)

Subseason	Resting Vigilant mean ± SD range	Resting mean ± SD range	Locomotion mean ± SD range	Feeding mean ± SD range	Grooming mean ± SD range	Other mean ± SD range
Early wet	48.9 ± 9.2	14.3 ± 12.8	14.9 ± 7.3	10.2 ± 5.1	3.6 ± 2.5	8.1 ± 2.5
	34.4–67.3	0.8–40.1	4.3–27.1	3.1–23.4	1.0–10.4	0–25.5
Late wet	54.1 ± 7.4	6.5 ± 7.4*	17.1 ± 6.5*	7.2 ± 6.5	5.1 ± 4.5	7.9 ± 12.6
	34.4–74.2	0.1–25.2	7.9–30.2	0.6–14.1	0.75–17.9	0–30.5
Early dry	55.6 ± 6.9	11.9 ± 8.0	10.5 ± 3.1	9.8 ± 4.6	5.1 ± 1.8	6.9 ± 5.4
	39.3–68.4	0.8–29.1	6.3–16.3	1.7–17.7	1.7–7.8	0.1–18.4
Late dry	55.5 ± 9.8	17.8 ± 7.9	6.3 ± 3.2	11.5 ± 4.6	3.6 ± 1.9	5.3 ± 6.9
	43.9–72.7	3.1–28.3	2.2–12.6	0.5–20.4	0.2–6.6	0–23.9

Percentages of behaviors were calculated for the time the individuals were clearly visible during nocturnal observations (401.47 h). *Indicates that the value is significantly different from those of the remaining seasons at level *P* < 0.05

Sex of the study subjects did not have a significant effect on time spent on Locomotion, Resting, Resting Vigilant, Feeding, and Grooming (LMM; Locomotion: χ^2^ = 0.92, df = 1, *P* = 0.34; Resting: χ ^2^ = 2.04, df = 1, *P* = 0.15; Resting Vigilant: χ ^2^ = 0.28, df = 1, *P* = 0.59; Feeding: χ ^2^ = 0.78, df = 1, *P* = 0.38; Grooming: LMM: χ ^2^ = 3.23, df = 1, *P* = 0.07). Sexes differed only in one behavioral category, with males spending more time on activities categorized as Other (LMM: χ ^2^ = 3.84, df = 1, *P* = 0.04) (see ESM Fig. S2 for more detailed apportionment of different behavioral categories between the sexes across all subseasons).

Subseason did not affect time spent feeding (LMM: χ ^2^ = 7.03, df = 3, *P* = 0.07), nor were there any seasonal influences on time spent Resting Vigilant (LMM: χ ^2^ = 4.84, df = 3, *P* = 0.18) or behaviors classed as Other (LMM: χ ^2^ = 2.31, df = 3, *P* = 0.51). We found a significant seasonal effect on Grooming (LMM: χ ^2^ = 7.96, df = 3, *P* = 0.04) but post hoc comparisons revealed no statistically significant differences between individual subseasons.

**Fig. 5:**
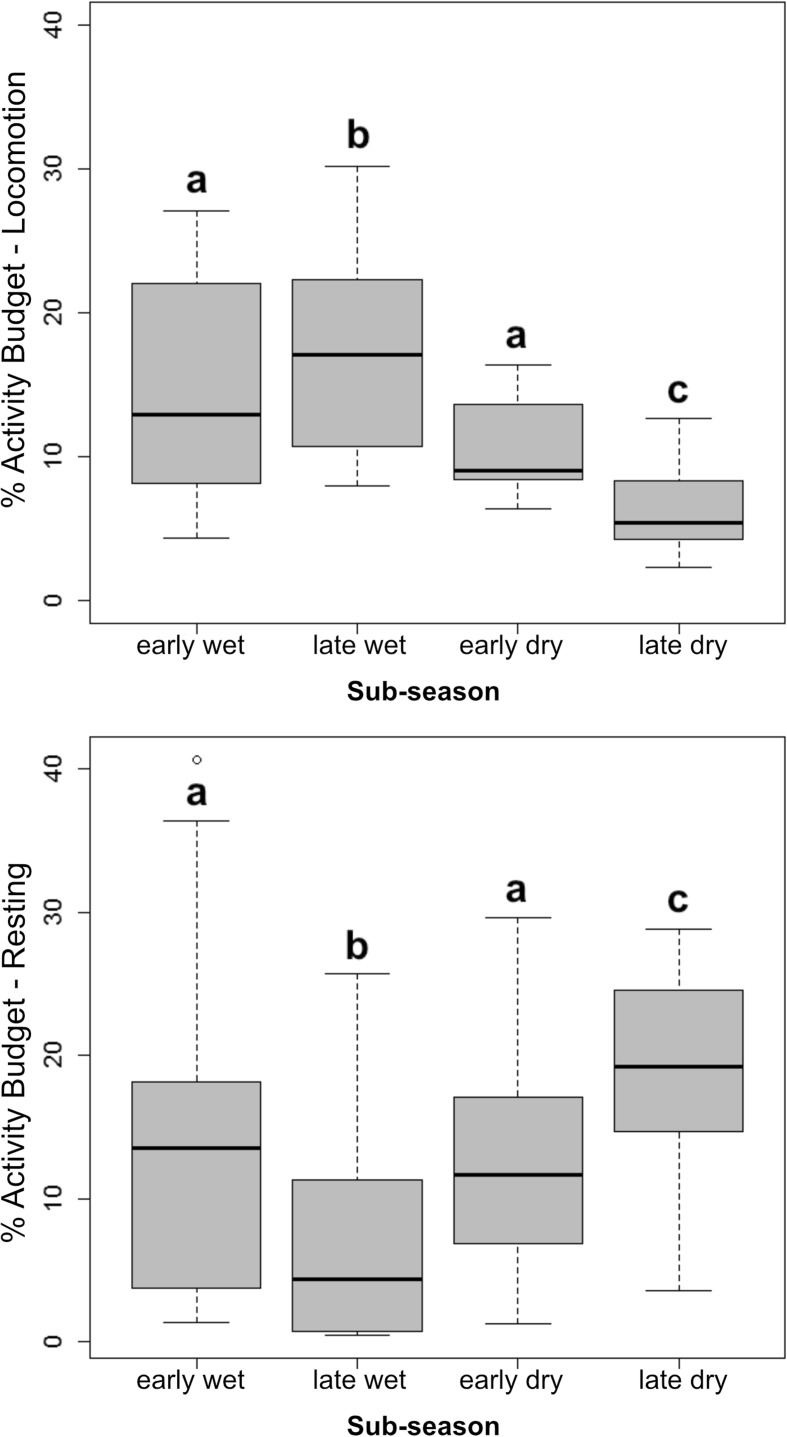
Percentage of time Sahamalaza sportive lemurs (*N* = early wet: 14; late wet: 14; early dry: 12; late dry: 11) in Ankarafa Forest, Sahamalaza National Park, northwest Madagascar, spent on locomotion and resting behaviour between October 2015 and August 2016 by subseason. Letters indicate statistically significant different values at *P* < 0.05. Black bars depict the median, the gray boxes the upper and lower quartiles, and whiskers the range.

Locomotion was significantly affected by subseason (LMM: χ ^2^ = 77.74, df = 3, *P* < 0.01): Tukey post hoc pairwise comparisons indicated that locomotion was highest in the late wet season compared to the rest of the year (late wet–early wet: *z* = 2.6, *P* = 0.4; late wet–early dry: *z* = −3.4, *P* < 0.01; late wet–late dry: *z* = −7.1, *P* < 0.01) while the study individuals spent the least amount of time with locomotion during the late dry season (late dry–early wet: *z* = −4.5, *P* < 0.01; late dry–early dry: *z* = −3.8, *P* < 0.01) (Fig. 5). Similarly, subseason had a significant effect on the time spent Resting (LMM: χ ^2^ = 22.57, df = 3, *P* < 0.01), and study individuals spent significantly less time resting in the late wet season than during any other period (late wet–early wet: *z* = −2.8, *P* = 0.02; late wet–early dry: *z* = 2.7, *P* = 0.03; late wet–late dry: *z* = 4.1, *P* < 0.01) (Fig. 5). The interactions between sex and subseason showed no statistical significance in any recorded behavior (Locomotion: LMM: χ ^2^ = 4.66, df = 3, *P* = 0.19; Resting: LMM: χ ^2^ = 1.01, df = 3, *P* = 0.79; Resting Vigilant: LMM: χ ^2^ = 0.74, df = 3, *P* = 0.86; Feeding: LMM: χ ^2^ = 1.51, df = 3, *P* = 0.67; Grooming: LMM: χ ^2^ = 0.74, df = 3, *P* = 0.86; Other: LMM: χ ^2^ = 1.77, df = 3, *P* = 0.62).

### Seasonal Effects on Home Range Size and Ranging

There was no difference in the size of the home ranges (overall size mean ± SD: 0.67 ± 0.53 ha, range: 0.22–1.4 ha) between the wet and the dry season (paired Student’s *t* test: *t* = −0.13, df = 10, *P* = 0.86). Overlap of the 50% core areas between the wet and the dry season was 26.8 ± 18.8% (mean ± SD, range: 7.6–67.9%).

**Fig. 6:**
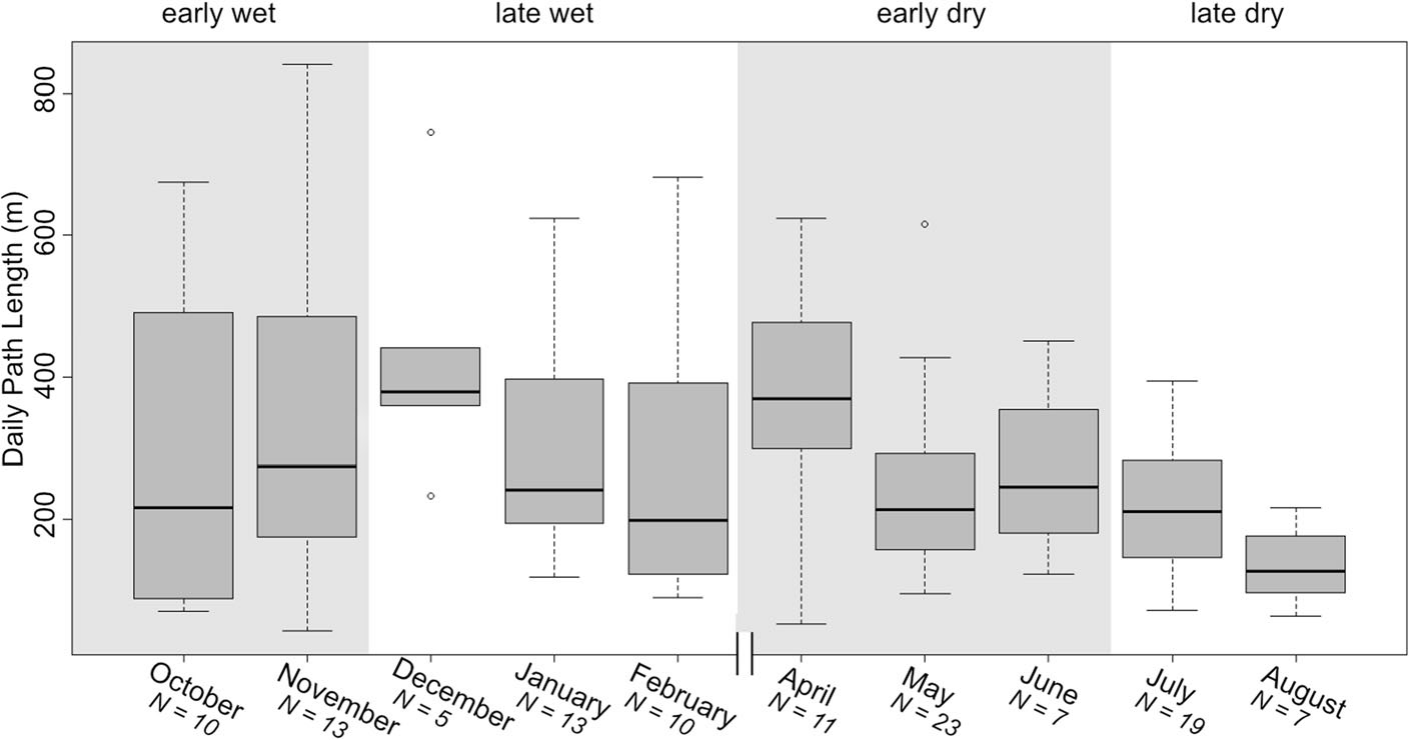
Daily path lengths of 14 Sahamalaza sportive lemurs (*N* = early wet: 14; late wet: 14; early dry: 12; late dry: 11 individuals), recorded in Ankarafa Forest, Sahamalaza National Park, northwest Madagascar between October 2015 and August 2016 by month of data collection and subseason. No data collection in March. Black bars depict the median, gray boxes the upper and lower quartiles, and whiskers the range.

Path length was not significantly affected by subseason (LMM: χ ^2^ = 2.16, df = 3, *P* = 0.53) or sex (LMM: χ ^2^ = 0.38, df = 31, *P* = 0.54) (Fig. 6). The effect of minimum temperature was nonsignificant (LMM: χ ^2^ = 2.7, df = 3, *P* = 0.09) and rainfall affected path length significantly (LMM: χ ^2^ = 4.66, df = 1, *P* = 0.03). Lemurs traveled shorted distances on nights with rainfall >5 mm than on nights with <5 mm.

### Seasonal Effects on Sleeping Site Choice

The visibility of the study individuals during the day varied between seasons (Table V). Sleeping sites were usually either liana tangles, cavities in dead and living trees, in palm leaves, or simply on branches. Some individuals used the same trees repeatedly year-round. Cavities in dead or living trees were used mainly during the colder months (I. Mandl *pers. obs*.). These cavities fulfilled a double function: the exposed entry hole enabled sunbathing, while the cavity was often deep enough for the individual to fully hide from predators. However, as we could not see individuals in their sleeping trees for 31.6% of the days we recorded them, we could not quantify the number of different sleeping site types.

**Table V:** Percentage of days on which radio-collared Sahamalaza sportive lemurs (*N* = 14) were visible/not visible during sleeping site checks in Ankarafa Forest, northwest Madagascar between October 2015 and August 2016, by subseason

Subseason	GPS points recorded	Not visible (%)	Visible (%)
Early wet	238	22.3	77.7
Late wet	287	45.3	54.7
Early dry	302	29.1	70.9
Late dry	175	26.9	73.1

GPS points are the locations of sleeping sites that we could determine. We found individuals classed as “not visible” using their radio-collars but could not see them clearly

**Fig. 7:**
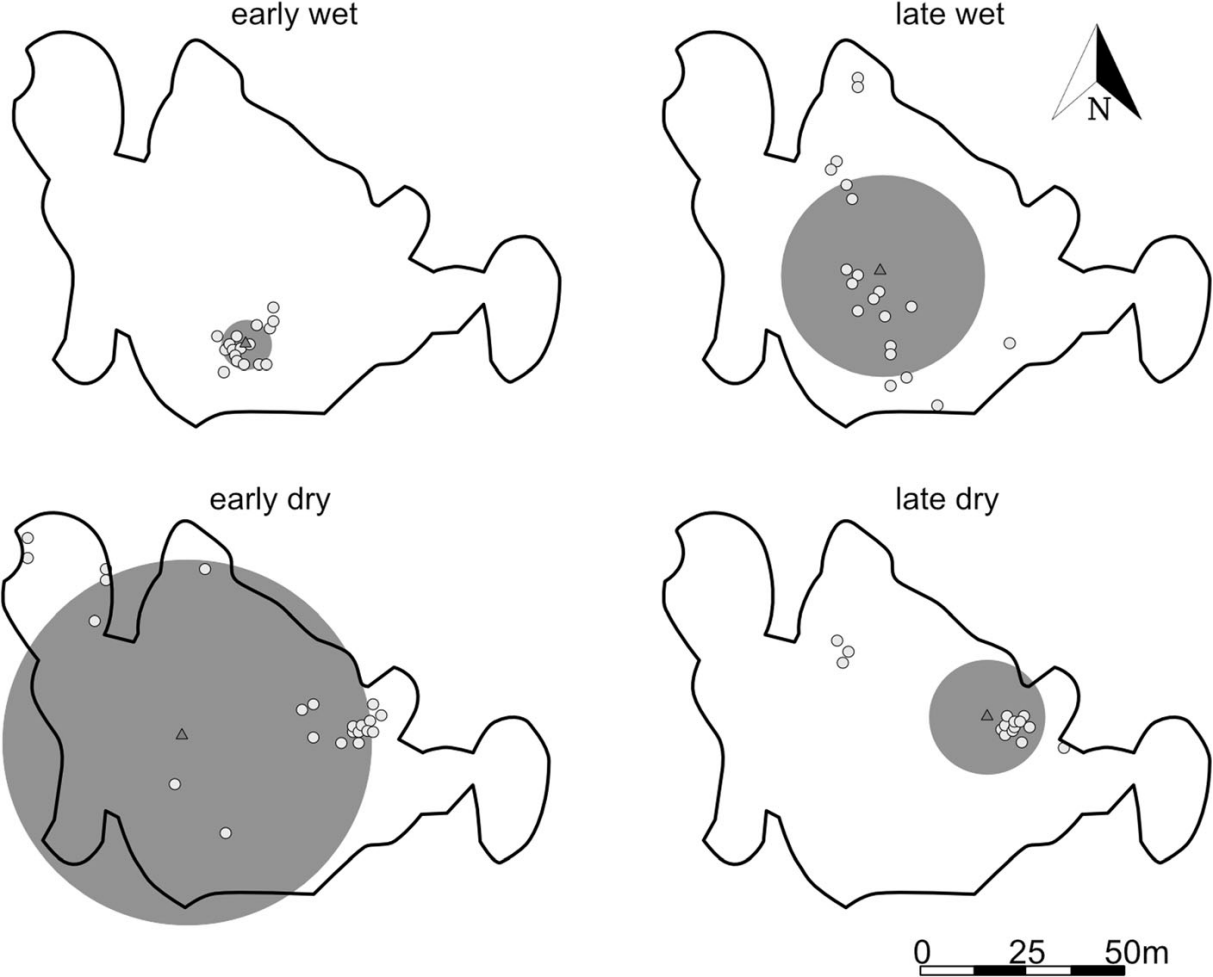
The location of sleeping sites in each subseason for one Sahamalaza sportive lemur (12 M) recorded in Ankarafa Forest, Sahamalaza National Park, northwest Madagascar between October 2015 and August 2016. The black outline represents the annual home range (99% KDE); gray dots individual sleeping site locations (*N* = early wet: 17, late wet: 21, early dry: 29, late dry: 16). Triangles illustrate the spatial centroids and the radius of the gray-shaded circles around the spatial centroids the mean standard distance for each subseason (early wet: 5.9 m; late wet: 23.6 m; early dry: 45.7 m; late dry: 15.8 m). Early wet = October/November/December; late wet = January/February; early dry = April/May/June; and late dry = July/August.

**Fig. 8:**
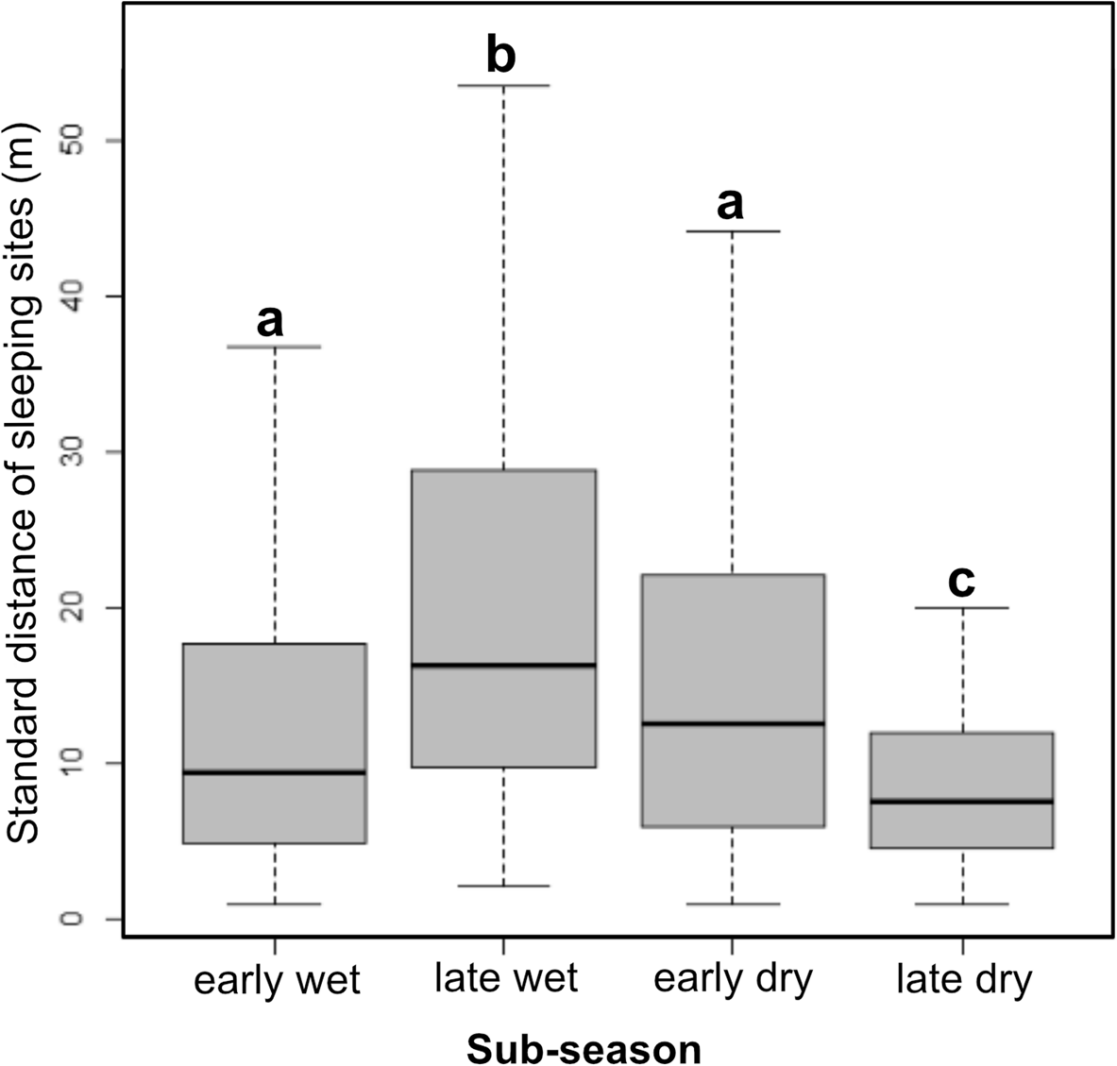
Distance between individual sleeping sites and mean sleeping site location for 14 individual Sahamalaza sportive lemurs (*N* = early wet: 14; late wet: 14; early dry: 12; late dry: 11) recorded in Ankarafa Forest, Sahamalaza National Park, northwest Madagascar between October 2015 and August 2016, for each subseason. Letters indicate statistically significant differences at *P* < 0.01. Black bars depict the median, gray boxes the upper and lower quartiles, and whiskers the range.

Over the study period, each study subject used multiple sleeping sites located in different areas of their home ranges (Fig. 7). The distance to the spatial centroid was affected by subseason (LMM: χ ^2^ = 99.43, df = 3, *P* < 0.01). Sites were further apart in the late wet (*z* = −9.7, *P* < 0.01) and early dry season (*z* = −6.7, *P* < 0.01) than in the late dry season (Tukey-adjusted, post hoc pairwise comparisons), when study individuals used sleeping sites in a limited area of their home range (Fig. 8).

## Discussion

Temperature and rainfall in the study region varied considerably over the study period of 10 mo, with the late dry season showing the lowest temperatures. Rainfall occurred mainly between October and March. Sportive lemurs showed few sex differences in behavior and only locomotive and resting behaviors differed significantly between subseasons. Time spent feeding increased over the dry season, but this was not statistically significant. There were no measurable differences in the sizes of home ranges between the dry and the wet season, but rainfall significantly negatively affected ranging behavior. While we could not record the number and types of sleeping sites, the locations of sleeping trees varied across subseasons, with wider spread of sleeping sites in the late wet season than the rest of the year.

### Seasonal Effects on Activity Budget

As we predicted, Sahamalaza sportive lemurs rested more and traveled less in the coldest period of the year. Across all seasons, Resting Vigilant made up nearly half of the activity budget, in line with previous studies that showed that this species remains stationary for prolonged periods at night (Seiler *et al.* 2014). However, time spent resting increased significantly in the coldest period.

These findings mirror what previous studies suggested to be a strategy for energy conservation during times of low resource abundance (e.g., Eppley et al. 2016; Ganzhorn *et al.* 2004; Nowack *et al.* 2013). The lemurs fed on the leaves of >40 different species of plants in previous studies (Seiler *et al.* 2014). During the present study, they added fruit to their diet in the wet season, but leaves constituted most of the diet year-round (I. Mandl *pers. obs*.) as observed in other folivorous primate species such as mantled howler monkeys (*Alouatta palliate*: Dunn *et al.* 2010), bushbabies (*Galago* spp.: Harcourt 1986), Cat Ba langurs (*Trachypithecus poliocephalus*: Hendershott *et al.* 2016), Japanese macaques (*Macaca fuscata yakui*: Hill 1997), diademed sifaka (*Propithecus diadema*: Irwin *et al.* 2014), black howler monkeys (*A. pigra*: Pozo-Montuy and Serio-Silva 2007), brown lemurs (*Eulemur fulvus*: Sato *et al.* 2014), and rhesus macaques (*M. mulatta*: Tang *et al.* 2016; van Schaik and Brockman 2005). More long-term studies on phenology and nutrition are needed to determine to what extent Sahamalaza sportive lemurs are subjected to resource fluctuations, but findings fail to support the idea that the coldest period signifies the environmentally most stressful time due to food limitations for red-tailed sportive lemurs (*Lepilemur ruficaudatus*: Ganzhorn 2002). White-footed sportive lemurs (*L. leucopus*) decreased activity levels in the coldest period of the year while leaves were still abundant and increased again with increasing ambient temperatures, when food availability reached a low point (Dröscher and Kappeler 2014). The authors suggest that minimum temperatures affected energy expenditure more than fluctuations in dietary resources. An increase in food intake, hypothesized to fuel heat production during the dry season, further points toward behavioral adaptations to cold temperatures (Dröscher *et al.* 2016). The sportive lemurs in our study increased the proportion of time spent feeding during the colder periods, but this effect was not statistically significant and data on resource availability are needed to conclude whether increased time spent feeding reflects a compensatory behavior for a diet of lesser quality (e.g., Hendershott *et al.* 2016) or a thermoregulatory strategy (e.g., Nowack *et al.* 2013). A further seasonal adaptation in feeding behavior is a change in nutrient composition of the diet: white-footed sportive lemurs increased nonprotein (e.g., fiber) intake during the dry season, possibly owing to variations in available foods (Dröscher *et al.* 2016). Diets high in fiber can affect heat production (Zhao and Wang 2007), further inducing thermoregulatory stress, but more research is needed to investigate the intertwined effects of high-fiber, folivorous diets and thermoregulation in primates.

A change in proportion of resting to traveling has been suggested to be a thermoregulatory strategy in other small, nocturnal primate species (Knox and Wright 1989; Schmid and Kappeler 2005). Behavioral thermoregulation to counteract cold stress can include a decrease in active behavior, positional changes (e.g., “hunched” posture while resting; Dagosto 1995) or social huddling during the day and night (Gilbert *et al.* 2010; Ostner 2002). During the present study, a female spent 3 h huddling with an unknown, but likely younger because smaller, individual during a particularly cold night, possibly to minimize heat loss, as in a wide range of endothermic animals (Gilbert *et al.* 2010; Kotze *et al.* 2008; Ostner 2002; Savagian and Fernandez-Duque 2017). Huddling effects physiological changes in mouse lemurs, *Microcebus murinus*, decreasing metabolic costs and thus conserving energy (Perret 1998). Sportive lemurs have low resting and basal metabolic rates, like other strepsirrhines (Bethge *et al.* 2017; Dorcas and Crompton 1998; Schmid and Ganzhorn 1996; Wright 1989, 1999). The sportive lemurs’ low metabolic rate and long resting bouts during the dry season may be in part due to physiological thermoregulation (Bethge *et al.* 2017; Kobbe *et al.* 2014; Kurland and Pearson 1986; Schülke and Ostner 2007; Sparrow and Newell 1998). However, more research is required to fully understand the effects of temperature fluctuations on sportive lemur physiology (see Bethge *et al.* 2017).

The sexes did not differ in the times they allocated to resting and traveling behaviors across the year. Overall males groomed less than females but engaged more in activities of the category “Other” owing to their increased vocal activity compared to females (*unpubl. data*). These results indicate that males and females did not face different energetic demands despite the additional requirements of lactation and gestation in females, in accordance with what has been found for white-footed sportive lemurs (Dröscher *et al.* 2016), ring-tailed lemurs (*Lemur catta*), and brown lemurs (*Eulemur* sp.: Simmen *et al.* 2010).

### Seasonal Effects on Ranging Behavior

We predicted a change in home range size between the warmer wet and colder dry seasons, reflecting decreased activity in the colder period of the year. The home range sizes recorded in this study were in line with what has been reported previously for this species (Seiler *et al.* 2015) but did not differ between the seasons. In addition, home range locations did not change considerably between the seasons, in contrast to those of primates that rely on fruiting trees year-round and change their ranges to incorporate available fruit (Garber 1993; Peres 1994; Wallace 2006). Home range size, and possibly location, differs between seasons if habitat requirements differ (e.g., Wiktander *et al.* 2001), which may not be the case for the folivorous Sahamalaza sportive lemur (Seiler *et al.* 2014).

The lemurs traveled shorter distances in nights with rainfall, which occurred mainly between December and February. Rainfall can affect the insulation properties of fur, reducing its thermal resistance (Webb and King 1984). The Sahamalaza sportive lemurs in this study may have sought to avoid getting wet by remaining in shelter during rain bouts. Variation in path length as a response to rising humidity levels has also been reported in other primate species: in gorillas (*Gorilla beringei*) shorter path lengths are associated with increase in humidity and rainfall (Ganas and Robbins 2005), whereas Javan slow lorises (*Nycticebus javanicus)* travel more in a more humid environment (Reinhardt *et al.* 2016).

The sex of the lemur did not affect path length, although the sexes have potentially different reproductive interests as males may roam to find receptive females (Lane *et al.* 2010). These findings reflect those for red-tailed sportive lemurs (Ganzhorn *et al.* 2004). Distance traveled also did not differ between the subseasons in this study: while individuals decreased the time spent in locomotion, they did not travel shorter distances. Shorter path lengths may reflect either a change in resource distribution (e.g., Hoffman and O’Riain 2011) or an adaptation to conserve energy in a climate with extreme temperatures (hot: e.g., Campos and Fedigan 2009; cold: Ganzhorn *et al.* 2004; Warren and Crompton 1997). Red-tailed sportive lemurs (Ganzhorn *et al.* 2004), red-ruffed lemurs (*Varecia rubra*: Vasey 2005), howler monkeys (*Alouatta caraya*: Raño *et al.* 2016), snub-nosed monkeys (*Rhinopithecus bieti*: Ren *et al.* 2009), and Phayre’s leaf monkeys (*Trachypithecus phayrei:* Carl 2009), all travel less in the coldest time of the year. Although heat production is generally fueled by locomotion, traveling can be energetically costly (Terrien *et al.* 2011), especially for species with low metabolic rates, such as sportive lemurs. However, the energetic costs of locomotive behavior in this genus have not been studied in depth (Dorcas and Crompton 1998; Warren and Crompton 1997). It is possible that colder temperatures have an additional constraining effect on primate movement, as heat may be lost through exposure of less insulated body areas (Paterson 1983). In contrast, white-faced capuchins (*Cebus capucinus*) reduce path length with increasing temperatures (Campos and Fedigan 2009). These primates reduced their travel distances and centred their activities around remaining water sources in hotter temperatures (Campos and Fedigan 2009). While the sportive lemurs we studied may also have been influenced by variation in resource distribution, the unequal sample sizes and variances in the data set did not allow for further analysis of path length. More detailed information is needed to determine the variability of environmental resources for this species.

### Seasonal Effects on Sleeping Site Choice

Based on previous results that the lemurs did not use tree holes after days with rainfall (Seiler *et al.* 2013), we predicted a difference in sleeping sites between subseasons. As the study subjects were hidden at their sleeping sites for at least a quarter of the days that GPS points were collected, we did not analyze the number and types of sleeping sites. A previous study indicated that microhabitats around sleeping sites are important to this species, suggesting only specific sleeping sites meet ecological needs (Seiler *et al.* 2013). The changes in sleeping site locations recorded in the present study may imply differing requirements or priorities (e.g., protection from rain) across the year. The lemurs used sleeping site locations that were spread over a greater area during the late wet season than the rest of the year. Lemurs may have sought more sheltered places during months with heavy rains, as suggested by Seiler *et al.* (2013): some of the tree cavities used during the dry season were very exposed and even collected water during the wet season (I. Mandl *pers. obs*.). This could explain the fact that the study individuals were least visible during sleeping site checks between December and February, the months with the highest rainfall, as they rested in well-covered day sleeping sites. However, this does not sufficiently explain why the study individuals slept in multiple locations spaced more widely apart, rather than staying in one or two well-sheltered sites.

Suitable sleeping sites may represent a limited resource, as suggested for Milne Edwards’ sportive lemurs, (*Lepilemur edwardsi*: Rasoloharijaona *et al.* 2003) and weasel sportive lemurs (*L. mustelinus*: Rasoloharijaona *et al.* 2010). The importance of high-quality sleeping sites that provide sufficient protection from predators and weather has been emphasized (Anderson 1998). Using sleeping sites far apart and changing them often can function in predator avoidance (Hrdy 1980; Smith *et al.* 2007): small-bodied primates such as tufted capuchins (*Sapajus apella*), moustached tamarins (*Saguinus imperator*), and Azara’s owl monkeys (*Aotus azarae*) change sleeping sites frequently and avoid sleeping in the same location on consecutive nights, presumably to prevent predators from anticipating sleeping site locations (di Bitetti *et al.* 2000; Savagian and Fernandez-Duque 2017; Smith *et al.* 2007). We did not assess the predation pressure on sportive lemurs by raptors (e.g., Madagascar harrier hawk, *Polyboroides radiatus*), snakes (e.g., boas, *Acrantophis madagascariensis*), or carnivores (e.g., fossa, *Cryptoprocta ferox*) in the study area but predation pressure was higher during the dry season than the rest of the year in previous studies conducted in similar habitats (Gursky and Nekaris 2007; Rasmussen 2005; Schnoell and Fichtel 2011). Our study subjects slept in a limited area of their home range during the late dry season (July and August), using only one or two sleeping sites repeatedly. They often rested in very exposed spots, as recorded by the high visibility in this period. Sahamalaza sportive lemurs were easily found, often resting exposed, during the dry season in a previous study that showed that this species was highly vigilant and reactive toward calls of predators, regardless of sleeping site type (Seiler *et al.* 2013). Sleeping sites chosen in the dry season may provide increased protection from predators by enabling earlier detection in a more exposed resting place (Gursky and Nekaris 2007).

The lemurs we studied may also use dry season sleeping sites based on other factors, such as ambient temperature: colder temperatures induce animals to choose sleeping sites that provide them with thermoregulatory benefits such as better insulation (Karanewsky and Wright 2015; Radespiel *et al.* 1998) or sun exposure. Azara’s owl monkeys face similar trade-offs and choose sleeping sites to minimize predation risk while being constrained by thermoregulatory requirements (Savagian and Fernandez-Duque 2017). These primates rest in more exposed places during colder periods, enabling sunbathing behavior—a possible parallel to Sahamalaza sportive lemurs, which remain active during the day, changing position frequently but not leaving their sleeping sites (Ruperti 2007; Seiler *et al.* 2013). Colder temperatures at night may have induced the lemurs to rest in exposed sites that allow for sunbathing to rewarm faster as in the marsupial fat-tailed antechinus (*Pseudantechinus macdonnellensis*: Geiser *et al.* 2002). Suitable, sun-exposed sleeping sites that provide protection from predators may be limited in the colder period of the year, which may explain why the lemurs returned to the same one or two locations during the late dry season.

The spread in sleeping site locations may have been influenced by social drivers: sleeping sites used during the late wet season, which coincides with the premating period, were furthest apart and located in multiple places within the home range, some especially close to the borders. During such periods, the study individuals may have sought to mark their territories by sleeping in and marking multiple locations (Day and Elwood 1999; Reichard 1998; Singhal *et al.* 2007).

In conclusion, Sahalamaza sportive lemurs showed seasonal changes in activity budgets and sleeping site locations, as well as fluctuations in travel distances across the year. The underlying drivers of seasonally changing behavior remain to be studied in detail. Future studies should also aim to understand variation in sportive lemur ecology, in particular 1) the physiological adaptations of this genus to strong temperature fluctuations, 2) the trade-off between predation pressure and thermoregulatory requirements, and 3) the nutrient requirements and feeding behavior across all season.

Our results reflect findings from studies of other primate taxa: seasonal changes in climate, and accompanying resource abundance, influence primate behavioral ecology (Hemingway and Bynum 2005). Primates display behavioral flexibility and energy-conserving behavior when faced with environmental stressors such as low food availability or temperature fluctuations (van Schaik and Brockman 2005). In view of expected long-term changes in global climate, research on the tolerance and degree of flexibility across primates is required to anticipate species’ reactions (Beever *et al.* 2017).

## Electronic supplementary material

Fig. S1 (DOCX 80 kb)

Fig. S2 (DOCX 318 kb)
