# Supplementary material for: The Effects of Climate Seasonality on Behavior and Sleeping Site Choice in Sahamalaza Sportive Lemurs, Lepilemur sahamalaza
Source: Int J Primatol. 2018 Sep 11;39(6):1039–67. doi: 10.1007/s10764-018-0059-1 (PMC6300582; doi:10.1007/s10764-018-0059-1)
Supplement: Supplementary file 1 — (DOCX 80 kb) [file 10764_2018_59_MOESM1_ESM.docx]

**Electronic Supplementary Material**

**The Effects of Climate Seasonality on Behavior and Sleeping Site Choice in** **Sahamalaza Sportive Lemurs, *Lepilemur sahamalaza***

**Isabella Mandl · Marc Holderied · Christoph Schwitzer**

**Fig. S1** Plots of cumulative home range size (in ha) over the number of nights each individual Sahamalaza sportive lemur, *Lepilemur sahamalaza*, was followed during behavioral observations between 2015 and 2016. GPS points were recorded for 6 h (18:00–00:00 h). Number above each plot indicates individual ID.
