# Supplementary material for: The Effects of Climate Seasonality on Behavior and Sleeping Site Choice in Sahamalaza Sportive Lemurs, Lepilemur sahamalaza
Source: Int J Primatol. 2018 Sep 11;39(6):1039–67. doi: 10.1007/s10764-018-0059-1 (PMC6300582; doi:10.1007/s10764-018-0059-1)
Supplement: Supplementary file 2 — (DOCX 318 kb) [file 10764_2018_59_MOESM2_ESM.docx]

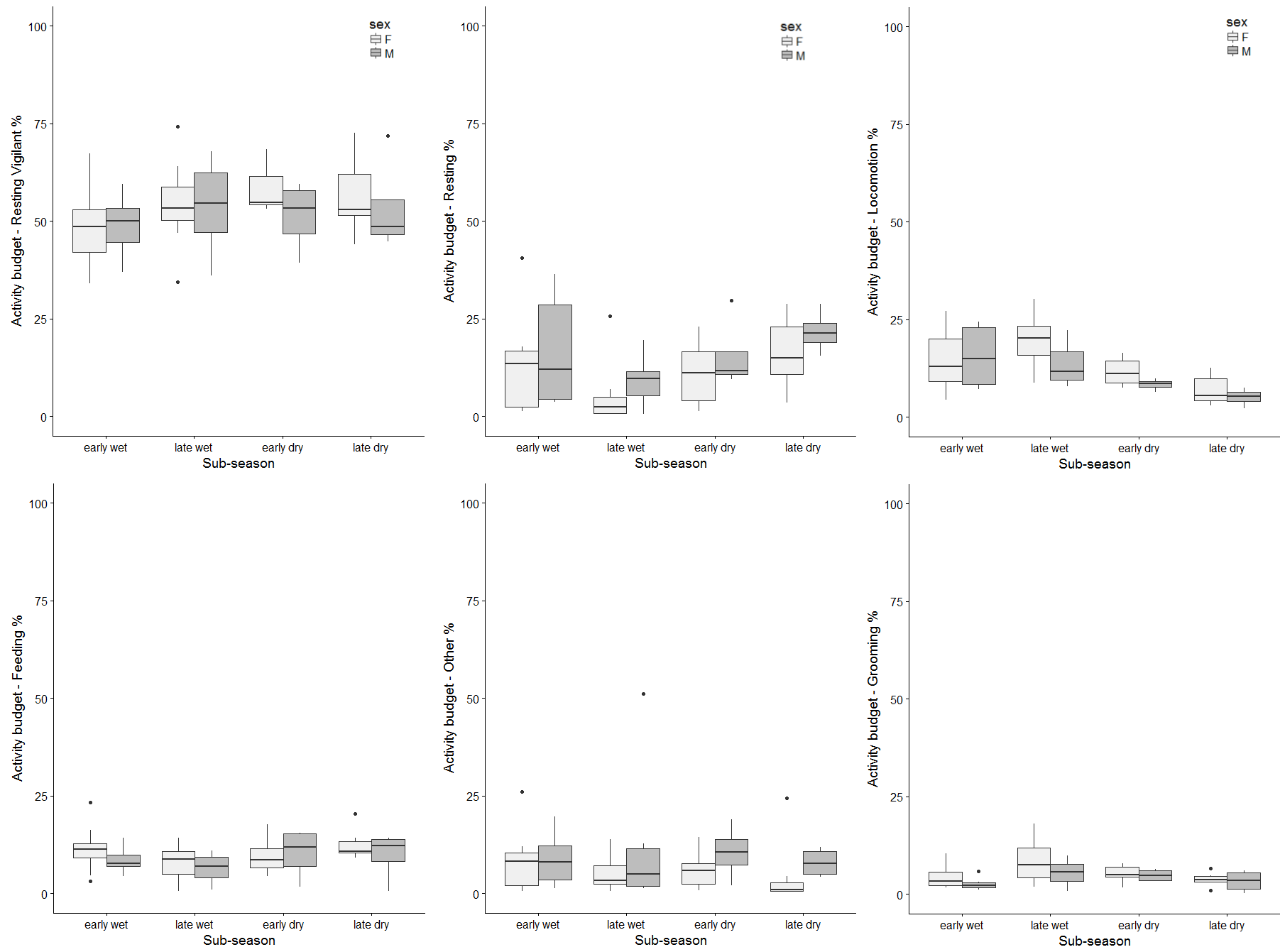


**Fig. S2** Detailed activity budgets (% of time spent on different behavioral categories) for both sexes of the studied Sahamalaza sportive lemurs, *Lepilemur sahamalaza*, across all subseasons (early wet: October–December; late wet: January–March; early dry: April–June; late dry: July–September). Sample size (number of individuals) varied between the subseasons: early wet: *N* = 14; late wet: *N* = 14; early dry: *N* = 12; late dry: *N* = 11. Black bars illustrate the median, gray boxes the upper and lower quartiles, and whiskers the range.
